# Relatedness facilitates cooperation in the subsocial spider, *Stegodyphus tentoriicola*

**DOI:** 10.1186/1471-2148-9-257

**Published:** 2009-10-27

**Authors:** Jasmin Ruch, Lisa Heinrich, Trine Bilde, Jutta M Schneider

**Affiliations:** 1 Zoological Institute and Museum, Biozentrum Grindel, University of Hamburg, Martin-Luther-King-Platz 3, 20146 Hamburg, Germany; 2 Department of Biological Sciences, Aarhus University, Ny Munkegade Building 540, 8000 Aarhus C, Denmark

## Abstract

**Background:**

Cooperative hunting and foraging in spiders is rare and prone to cheating such that the actions of selfish individuals negatively affect the whole group. The resulting social dilemma may be mitigated by kin selection since related individuals lose indirect fitness benefits by acting selfishly. Indeed, cooperation with genetic kin reduces the disadvantages of within-group competition in the subsocial spider *Stegodyphus lineatus*, supporting the hypothesis that high relatedness is an important pre-adaptation in the transition to sociality in spiders. In this study we examined the consequences of group size and relatedness on cooperative feeding in the subsocial spider *S. tentoriicola*, a species suggested to be at the transition to permanent sociality.

**Results:**

We formed groups of 3 and 6 spiders that were either siblings or non-siblings. We found that increasing group size negatively affected feeding efficiency but that these negative effects were reduced in sib-groups. Sib groups were more likely to feed cooperatively and all group members grew more homogenously than groups of unrelated spiders. The measured differences did not translate into differential growth or mortality during the experimental period of 8 weeks.

**Conclusion:**

The combination of our results with those from previous studies indicates that the conflict between individual interests and group interests may be reduced by nepotism and that the latter promote the maintenance of the social community.

## Background

A so-called 'tragedy of the commons' [1] arises when groups of animals or humans exploit a common resource and the selfish actions of individuals negatively affect group performance [2]. The extent of negative effects can range from a small reduction of benefits up to a point where the effects are detrimental to all members of the group. Examples include the formation of fruiting bodies in microbes [3], slime moulds [4], and the overexploitation of public goods, such as the environment [5] and the global climate by humans [6].

Social spiders create a common good if they communally hunt a large prey item that they digest as a group [7]. Cooperative hunting and foraging is rare in spiders and if it occurs, it is restricted to family groups [8-10]. Genetic relatedness but not familiarity has been found to reduce the tragedy of the commons among group feeding periodically social *Stegodyphus lineatus *spiders, supporting the view that kin selection is an important force in the evolution of sociality in these spiders [7]. The finding that cooperating with genetic kin reduces the disadvantages of within-group competition provides support for the hypothesis that high relatedness is a crucial step in the transition to sociality in spiders [10], as controversially discussed for the eusocial insects [11-13]. Genetic analyses have shown that permanent social spider colonies can be described as clones, with no or little genetic variation within and no gene flow among colonies [14-16]. This pattern arises because all permanently social spider species regularly inbreed [10]. Regular inbreeding is an evolutionary paradox because it removes all advantages associated with sexual reproduction and recombination [17] while the risks and costs [18] are largely retained. These costs of sex may be diminished through a primary sex ratio shift towards females, which is another convergent trait of all social spiders [10,19]. Strong benefits of genetic similarity may offset at least part of the costs. In the genus *Stegodyphus*, three species are social, and the remaining 14 species are periodically social [20,21]. The behaviour and ecology of only one of the periodically social species, *S. lineatus*, has been studied yet [22-24]. A recent molecular phylogeny suggests that *S. lineatus *is separated from the other species [20] and supports that the latter form three subgroups around the permanently social species that evolved independently within the genus [21]. To gain insights about evolutionary pathways towards sociality and inbreeding, the study of social species and their subsocial closely related sister species may be a particularly fruitful approach.

*S. tentoriicola *is sister to the social *S. dumicola *[20,21] and unpublished observations suggest that its social juvenile phase is extended in comparison to *S. lineatus *[10]. A field study revealed that rates of polyandry - female mating with multiple sires - are lower than in the congener *S. lineatus *which indicates that relatedness within broods is relatively increased [25]. This implies that sibling groups meet the expectations for high genetic similarity required to evolve cooperation [12]. Here we investigated whether genetic relatedness reduces the tragedy of the commons in *S. tentoriicola*. We compared cooperation during group feeding in genetically similar and dissimilar groups of juveniles from *S. tentoriicola*, using a protocol previously applied to *S. lineatus *[7]. In *S. lineatus*, Schneider and Bilde [7] showed that genetic similarity and not familiarity among groups members carried benefits of cooperation. It is a parsimonious assumption that similar mechanisms apply in *S. tentoriicola *and that familiarity does not play a role. We tested the effect of group size on benefits of kin cooperation by comparing feeding efficiency and growth of groups of two different sizes with singly feeding spiders. We ask whether the size of the group per se and the size of the feeding group (the members actually participating in the feeding event) play a role in determining feeding efficiency, and we examine how relatedness interacts with the dynamics of cooperation and competition by investigating the performance of the group in terms of feeding efficiency, growth and mortality. This study design allows us to assess the generality of previous findings on the existence of a social dilemma in communally feeding spiders, and on the role of nepotism for the resolution of this dilemma. This has implications for understanding the evolution of sociality in spiders.

## Results

### Effect of group size on feeding, growth and mortality

Generally, not all group members fed when a single prey item was introduced into their web. Hence, in groups of 6 spiders, on average three spiders shared a prey (3.3 ± 0.21 spiders feeding, n = 20), while in groups of 3 spiders mostly two or one spider fed (mean ± SD: 1.6 ± 0.16 spiders feeding, n = 22, One-factorial test: χ^2 ^= 19.76, DF = 1, p ≤ 0.0001). Below, we will refer to small (total group size = 3) and large foraging groups (total group size = 6), respectively.

Within a feeding period of two hours, prey items lost weight and this weight loss was an increasing function of the number of spiders that fed on it: large groups extracted 14.79 ± 7.55 mg (n = 20) from a fly; small groups extracted 11.98 ± 6.90 mg (n = 22); single spiders extracted 6.69 ± 4.13 mg (n = 11) (Kruskal-Wallis-Anova: χ^2 ^= 26.26, DF = 2, p ≤ 0.0001, Tukey-Kramer HSD, p ≤ 0.05, significant difference across all groups [large groups, small groups, single spiders]). The values are means over the repeated trials per group.

Even though large groups extracted more than small groups or singles, the mean per capita induced weight loss decreased with increasing groups size: single spiders extracted the most out of the prey (9.95 ± 0.85 mg, n = 11) and large groups the least (three: 7.22 ± 0.73 mg, n = 22, six: 5.10 ± 0.32 mg, n = 20) (Kruskal-Wallis-Anova: χ^2 ^= 23.13, DF = 2, p ≤ 0.001, Tukey-Kramer HSD, p ≤ 0.05, significant difference across all groups [large groups, small groups, single spiders]). However, this difference did not translate into significantly different growth (relative mass increase over observation period of 8 weeks, single: 0.35 ± 0.05, n = 10, three: 0.28 ± 0.02, n = 19, six: 0.32 ± 0.17, n = 19, Kruskal-Wallis-Anova: χ^2 ^= 1.88, DF = 2, p = 0.39). We included trial number as random factor into a general linear mixed model (GLMM) and tested the influences of total group size as well as feeding group size on feeding efficiency (Table 1). Interestingly, the significant effect of total group size arises from a significantly higher feeding efficiency in small groups as compared to singles and large groups (Tukey-Kramer HSD, p ≤ 0.05). The reason for this likely is the frequent feeding of single individuals as well as the advantage in hunting time that may balance out disadvantages in feeding efficiency as long as groups are very small.

**Table 1:** Influence of group size, sum of feeding spiders and trial number on feeding efficiency.

**source**	**F**	**DF**	**p**
Size of feeding group	103.7	1	≤ 0.0001
Group size (1, 3, 6 spiders)	7.14	2	0.001
Trial number			0.003

General linear mixed model (REML): trial number (random effect; t = 3.1), (whole model, r^2 ^= 0.59).

Mortality was significantly lower in single spiders than in groups (single: 7.7 ± 7.7%, n = 13, three: 9.1 ± 5.5%, n = 22, six = 13.3 ± 5.2%, n = 20, Kruskal-Wallis-Anova: χ^2 ^= 6.1, DF = 2, p = 0.047, Tukey-Kramer HSD, p ≤ 0.05, no significant differences between the groups).

### Effect of genetic relatedness on feeding, growth and mortality

The influence of relatedness on feeding performance was analysed separately for the two different group sizes (small and large). In this analysis, feeding events of single individuals were excluded.

#### Small groups

Only the number of feeding animals per trial explained a significant proportion of the mass extracted from a prey item in small groups (Table 2). The larger the feeding group, the more mass did the fly lose. Relatedness and trial number and their interaction were not significant. The number of spiders that participated in prey-sharing did not differ between sib and non-sib groups (One-factorial-test: non-sibs: 1.57 ± 0.23 spiders (n = 10), sibs: 1.71 ± 0.23 spiders (n = 12), χ^2 ^= 0.27, Z = -0.49, DF = 1, p = 0.6). Mass increase of the spiders over the experimental period did not differ between treatments (Table 4A). Within-group mortality was not influenced by treatment (One-factorial-test: non-sibs: 16.67 ± 11.39%, n = 10, sibs: 2.78 ± 2.78%, n = 12, χ^2 ^= 0.78, DF = 1, p = 0.37).

**Table 2:** Influence of treatment, sum of feeding spiders and trial number on feeding efficiency in groups of 3 spiders.

**source**	**F**	**DF**	**p**
Size of feeding group	15.4	1	0.0002
Treatment	0.66	1	0.42
Treatment × trial number	0.07	1	0.80
Trial number (random effect)			0.23

GLMM (REML): trial number (random effect; t = 1.49), (whole model, r^2 ^= 0.20).

**Table 3:** Influence of treatment (non-sib/sib) on relative per capita mass increase, mean body weights within groups before and after the experiment, and CV on body weights before and after experiment in groups of 3 (A) and 6 (B) spiders

	**non-sibs**	**sibs**	**statistics**	**p**
**A Groups of 3 spiders**				
Relative mean mass increase	0.39 ± 0.09 (9)	0.30 ± 0.03 (12)	Z = 0.75	0.46
(1 extreme outlier removed)	0.31 ± 0.03 (8)	0.30 ± 0.03 (12)	Z = 0.35	0.73
Mean body weights of groups before experiment [mg] (± SE mean)	4.65 ± 0.55 (10)	3.57 ± 0.24 (12)	t = -1.9	0.07
Mean body weights of groups after experiment [mg] (± SE mean)	14.42 ± 1.90 (9)	15.05 ± 2.16 (12)	t = -0.45	0.66
CV before experiment (± SE mean)	0.08 ± 0.014 (10)	0.07 ± 0.009 (12)	Z = 0.23	0.82
CV after experiment (± SE mean)	0.28 ± 0.080 (8)	0.16 ± 0.024 (12)	Z = 1.12	0.26

**B Groups of 6 spiders**				
Relative mean mass increase	0.33 ± 0.02 (10)	0.31 ± 0.03 (9)	t = -7.73	0.48
Mean body weights of groups before experiment [mg] (± SE mean)	3.63 ± 0.19 (10)	4.01 ± 0.38 (10)	t = 0.93	0.37
Mean body weights of groups after experiment [mg] (± SE mean)	11.10 ± 0.55 (10)	13.37 ± 0.85 (9)	t = 2.40	0.028
CV before experiment (± SE mean)	0.099 ± 0.01 (10)	0.067 ± 0.01 (10)	t = -2.47	0.024
CV after experiment (± SE mean)	0.33 ± 0.04 (10)	0.19 ± 0.02 (9)	t = -2.21	0.041

Means and SE are given and sample sizes in brackets (non-parametrical Wilcoxon test (Z) or parametrical t-tests (t) were performed).

#### Large groups

**Figure 1:**
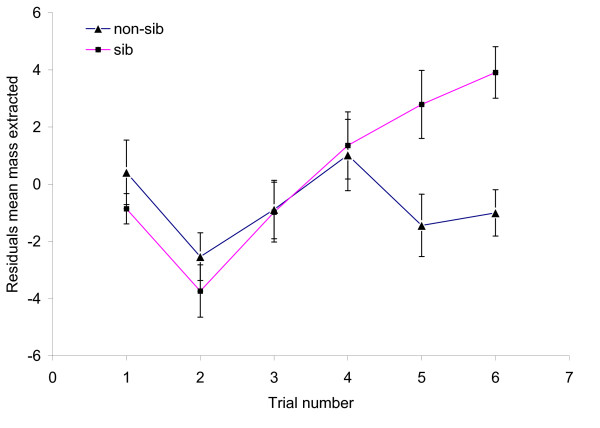
**Effect of relatedness in large groups on mean extracted prey mass (shown as residuals on feeding group size)**.

Large groups extracted more from a prey item when they were sibs rather than non-sibs. A GLMM showed that, apart from trial number and the size of the feeding group, relatedness had a significant effect on feeding efficiency (Table 4). The significant interaction between trial number and treatment resulted from a stronger increase in the mass extracted over sequential trials in the sib groups than in the non-sib groups (Figure 1).

**Table 4:** Influence of treatment, sum of feeding spiders and trial number on feeding efficiency in groups of 6 spiders.

**Source**	**F**	**DF**	**P**
feeding group size	76.4	1	≤ 0.0001
Treatment	5.4	1	0.023
Trial number			0.0003
Trial number × treatment	6.4	1	0.013
Trial number × sum of feeding spiders	5.3	1	0.024
Treatment × sum of feeding spiders	1.4	1	0.24

GLMM (REML): trial number (random factor; t = 3.95), (whole model, r^2 ^= 0.68).

**Figure 2:**
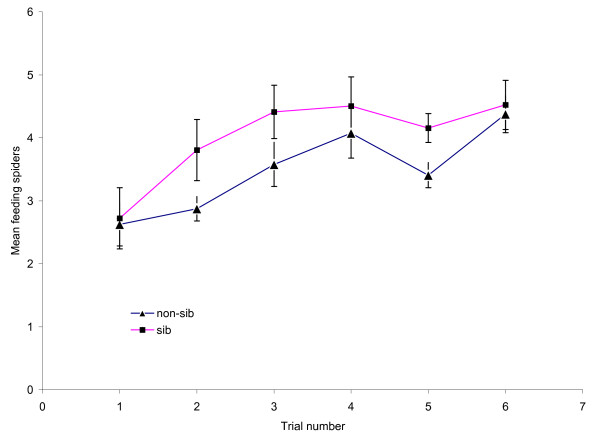
**Effect of relatedness in large groups on mean number of feeding spiders per trial**.

Sib groups fed in consistently larger numbers than non-sib groups (Figure 2) and a GLMM on the size of the feeding group revealed a significant treatment effect (F_1,97 _= 5.65, p = 0.02) in addition to a significant positive effect of trial number (random effect t = 4.51, p = 0.0001). The model explained 22% of the variation in the size of the feeding group.

Relative mass increase of spiders did not differ significantly between treatments (Table 3B) although the final mass of sib groups was significantly higher than in non-sib groups. However, sib groups started with a slightly although not significantly higher mass (Table 3B).

Spiders in the sib treatment grew more homogeneously than spiders in groups with non-sibs (CV Table 3B): Coefficients of variation in body weights of spiders within groups (CV_bm_) already differed at the start of the experiment (Table 3B) so that we included CV_bm _at the start as a covariate into the model. An ANCOVA on CV_bm _of groups of both sizes at the end of the experiment revealed that independent of the CV_bm _at the beginning of the experimental period, the CV_bm _in groups of non-sibs at the end of the experiment was significantly higher than in sib groups and significantly higher in large groups than in small groups (Table 5).

**Table 5:** Influence of initial CV, initial number of spiders, and Treatment, on CV at the end of the experimental period

**Source**	**F**	**p**
CV at start	0.09	0.89
Initial number of spiders	5.21	0.03
Treatment	8.19	0.007
Initial number of spiders × treatment	1.89	0.18

ANCOVA: One outlier had to be removed to achieve a normal distribution of the residuals (whole model, r^2 ^= 0.33, F_3,34 _= 5.47, p = 0.004). Inclusion of the outlier removes the significance of the factor "initial number of spiders" but does not change qualitative results of the other factors.

Treatment had no influence on within-group mortality (One-factorial test: non-sibs: 11.67 ± 4.34%, n = 10, sibs: 15.00 ± 9.76%, n = 10, χ^2 ^= 0.21, DF = 1, p = 0.63).

## Discussion

We experimentally investigated feeding efficiency, growth and mortality in groups of different sizes in the subsocial spider *S. tentoriicola *and found that relatedness reduced the negative effects of feeding competition in large groups. Feeding competition is a consequence of communal hunting and feeding, and is typical of social spiders [26], as of group living animals in general [27]. While competition is an inevitable cost of group living, cooperation among kin may negate this cost [7]. Here, this effect was apparent when groups were large, indicating a dynamic cost-benefit relationship between competition and cooperation shaped by the degree of competition (group size) and genetic composition of the group [27].

In *S. tentoriicola*, increased group size had a strong negative effect on feeding efficiency of individuals. Even though large groups extracted more prey mass than small groups or single spiders, individuals within large groups did not grow better. On the contrary, the per capita extracted prey mass decreased with increasing group size: single spiders fed significantly more efficiently than groups of three and six spiders. Thus, group feeding imposes a cost. Our results are consistent with the finding that feeding in a group reduces prey mass taken out per unit time in social *Stegodyphus mimosarum *[22]. These results indicate that communal feeding in spiders generally involves a social dilemma.

Spiders have to compete over digestive investment because of their feeding mode. They all inject their digestive enzymes into the prey and suck the liquidized content out of the exoskeleton [28]. Digestive enzymes are costly to produce [29] and hence, some spiders may benefit by consuming food digested by enzymes of a conspecific without providing investment themselves. Individuals should then hold back their enzymes and invest smaller portions that will not digest more than the donor itself can ingest. This process will reduce the speed of prey digestion in relation to the number of feeders. The more individuals occupy feeding positions on the prey carcass the more likely can neighbouring spiders benefit from the investment of others. With increasing group size cheating by holding back investment into digestive liquids should increase in frequency. This may explain the effect of group size found in our study and in a previous study on social *S. mimosarum *[30]. This resembles a classical dilemma termed tragedy of the commons because selfishness negatively affects the performance of the group but cannot be easily given up by individuals [2]. Feeding among kin may provide a solution to the tragedy of the commons through benefits of kin cooperation as seen here. The benefit of relatedness in this study was only large enough to be detected when three or more spiders regularly fed together. Hence, the effect of relatedness may be increasingly relevant in balancing competitive costs above a certain level of competition [31]. The relevance of such a compensation of costs of feeding competition is apparent from an experiment with social spider colonies that produced relatively more reproductive females if lipid rich prey was supplemented [32].

The result that relatedness reduces the negative effects of competition is consistent with previous findings in a related species [7]. Groups consisting of siblings had a higher feeding efficiency, suggesting a reduced inclination to cheat or an increased motivation to invest in the digestive process at the risk of the participation of other feeders. We did not employ a cross-fostering design in this study and hence have no experimental confirmation that genetic similarity and not familiarity is responsible for the observed effects. However, it is a parsimonious assumption that related species do not differ in such major traits.

In contrast to the observations on *S. lineatus*, sibling groups showed a larger feeding group size than non-sibling groups. In communally feeding spiders, dominant (large) individuals may be able to monopolize prey, while subordinate (small) individuals consistently succumb [26] which may lead to an unbalanced growth of the group and mortality due to starvation among the smaller individuals [8]. In our study, large groups of sibs shared prey with more conspecifics than non-sibs and sibs grew in a more homogeneous manner, resulting in a lower coefficient of variation at the end of the experiment. In non-sib groups, few individuals gained much weight while others hardly grew.

Despite the larger feeding group sizes, sib groups still performed better in feeding efficiency compared with non-sib groups in terms of more successful extraction of prey mass. However, the more successful extraction of prey mass did not result in higher growth rates, as found in *S. lineatus *[7]. Perhaps such a difference would have become apparent had our study run over a longer time period. This may also explain why - unlike in *S. lineatus *[7] where a marginally significant reduced mortality in sib groups was found - mortality did not differ between treatments in this study and was generally very low, although single individuals survived better than individuals in groups. We did not observe cannibalism which was found in *S. lineatus *under starvation [31].

*Stegodyphus *spiders represent one of the few study systems of the evolution of cooperation with convincing empirical evidence for genuine kin discrimination as opposed to nest-mate recognition [7,33]. Here we further corroborate the potential for kin discrimination in spiders, as our observations suggests that kin recognition is involved in producing the differences in cooperative feeding [7,8]. Kin recognition could affect higher motivation to invest and share costly enzymes in *S. tentoriicola*, whereas non-sibs may save digestive enzymes rather than sharing them. Alternatively, differences in feeding efficiency might be attributable to a higher compatibility of digestive enzymes among siblings compared to non-siblings. However, this hypothesis does not account for the increased inclination to forage communally of siblings obvious from the consistently larger feeding groups among siblings. After matriphagy, *S. tentoriicola*-spiderlings live, hunt and feed communally over an extended period which is reported to be longer than in *S. lineatus*. The latter species does not show the same behaviour and sib and non-sib groups did not show different feeding group sizes [7].

## Conclusion

Stronger cooperation within broods might promote prolonged associations of juveniles in *S. tentoriicola *and may underlie the loss of pre-mating dispersal apparent in the permanent social spiders [34], especially when the costs of dispersal are high [35]. Once pre-mating dispersal is lost it leads to an inbreeding mating system [10]. Through increasing within-group relatedness inbreeding might in turn favour the evolution of further kin-selected cooperative traits [36], thus generating a positive feedback that might culminate in the evolution of permanent group living or eusociality [7,37].

## Methods

### Study animals

*Stegodyphus tentoriicola *(Purcell, 1904) is an eresid spider distributed in South Africa [21]. It is a semelparous species, i.e. females invest all resources in a single clutch, a trait likely common to all species of the genus [38]. Female spiders care for their brood and feed the spiderlings via regurgitation before they are finally consumed by their offspring (matriphagy) [39]. The spiderlings cooperate in foraging and web building even after their mother has died [8].

The spiders used in the experiment are offspring of females that were collected in April 2008 from their natural habitat near Cradock (South Africa, 32°10' S 25° 37' E). They hatched between April and May 2008. The hatched juveniles and their mothers were left inside their natal nests and were kept in plastic containers in the laboratory. Temperature ranged between 20° - 28°C and a ventilator provided airflow.

After matriphagy, in June 2008, spiderlings of eight clutches were randomly assigned to four treatments in a 2 × 2 factorial design, with the following group compositions: (i) 3 sibs (n = 12); (ii) 6 sibs (n = 10); (iii) 3 non-sibs (n = 10); or (iv) 6 non-sibs (N = 11). All individuals in non-sib groups were derived from different mothers. Spiderlings were weighed to the nearest 0.1 mg by using an electronic balance and placed into Petri dishes (5.5 cm) either in groups of three (n = 22) or six (n = 21) animals. In addition, we separated 13 spiderlings which were placed individually into Petri dishes.

As soon as the spiderlings had produced silk in their Petri dishes, the feeding experiment began. A CO_2_-anesthetized *Calliphora *fly was weighed to the nearest 0.1 mg and put into the Petri dish. After the first spiderling attacked the fly, we determined the number of feeding spiderlings every 15 minutes for two hours (9 checks). When the prey was not attacked within 30 minutes, the trial was interrupted and the group was not used until the next feeding interval. Therefore, sample sizes can differ depending on the factors analysed. After two hours, the fly was removed and weighed again. It is important to note that the fly was only to a small part digested after this period. Hence single spiders did not benefit from having a whole fly while the others got only a part. For each group, we calculated the mean extracted prey mass in relation to the mean number of feeding spiderlings (mean over the 9 checks). In all treatments the time between feeding trials was the same (one week, except for the interrupted trials). Additionally, the spiderlings were fed once a week with one *Drosophila *per capita, i.e. all spiders had the same amount of food available. All spiderlings were weighed again at the end of the experiment. Data analyses were performed with JMP 7. For the analyses of feeding efficiency related to size of the feeding group, we excluded all incidences where only a single spider had fed. We used non-parametric rank based tests (Wilcoxon test) or one-factorial test based on a χ^2 ^distribution when the assumptions for parametric tests were not met. We computed general mixed models (GLMM) using restricted maximal likelihood (REML) with trial number as random factor. JMP 7 does not compute F-values but t-values for random factors.

## Authors' contributions

JMS and TB conceived and designed the experiments. JR and LH conducted the experiments. JR and JMS analysed the data and wrote the ms.
